# Tranexamic Acid for Hyperpigmentation Disorders: A Literature Review on Efficacy and Safety in Melasma and PIH

**DOI:** 10.1111/jocd.70692

**Published:** 2026-01-28

**Authors:** Ahmed AlJabr, Aseel Muhana I. AlAnazi, Rakan Abdulkarim A. AlEtebi

**Affiliations:** ^1^ Habib Medical Center Riyadh Saudi Arabia; ^2^ College of Medicine Dar Al Uloom University Riyadh Saudi Arabia

## Abstract

**Background:**

Hyperpigmentation disorders, including melasma and post‐inflammatory hyperpigmentation (PIH), are common dermatologic conditions associated with significant cosmetic and psychological burden. Tranexamic acid (TXA), an antifibrinolytic agent, has gained increasing attention due to its anti‐inflammatory and antimelanogenic properties.

**Objective:**

To review the current evidence on the efficacy and safety of tranexamic acid in the management of hyperpigmentation disorders, particularly melasma and PIH.

**Methods:**

A narrative literature review was conducted using PubMed, Scopus, and Google Scholar to identify clinical and observational studies evaluating the efficacy, safety, and comparative outcomes of oral, topical, and intradermal tranexamic acid in hyperpigmentation disorders. Data were descriptively analyzed with comparison to standard treatments such as hydroquinone and laser therapies.

**Results:**

Evidence from randomized and comparative studies demonstrates that oral, topical, and intradermal TXA significantly reduce pigmentation indices and improve quality‐of‐life scores in patients with melasma and PIH. Oral TXA at doses of 250–500 mg twice daily showed sustained clinical improvement with minimal adverse effects, most commonly mild gastrointestinal symptoms and menstrual irregularities. Topical and intradermal formulations demonstrated comparable or superior efficacy to hydroquinone with fewer irritant reactions. Combination therapies (e.g., TXA with hydroquinone or laser) yielded enhanced and longer‐lasting outcomes.

**Conclusion:**

Tranexamic acid represents a promising and well‐tolerated therapeutic option for hyperpigmentation disorders. Its efficacy across multiple administration routes, favorable safety profile, and synergistic potential with existing therapies support its expanding role as both a primary and adjunctive treatment in dermatologic pigment management.

## 1 Introduction

Among the most prevalent and cosmetically upsetting dermatological conditions worldwide are hyperpigmentation diseases, which include melasma, Riehl's melanosis, post‐inflammatory hyperpigmentation (PIH), lichen planus pigmentosus, and others [1]. In addition to affecting physical appearance, these conditions frequently result in psychological discomfort, a worse quality of life, and social shame.

Although treatment approaches include a variety of topical medications (hydroquinone, retinoids, azelaic acid, kojic acid), chemical peels, and laser/light‐based procedures, each technique has drawbacks [2]. Over the past 10 years, tranexamic acid (TXA) has drawn a lot of attention in dermatology due to its depigmenting qualities. By preventing plasminogen from being converted to plasmin, which is involved in the breakdown of fibrin, tranexamic acid is known to reduce bleeding through its antifibrinolytic action. Because of its strong plasmin inhibitory action, TXA is believed to have anti‐inflammatory, antimelanogenic, and antiangiogenic properties in addition to its antifibrinolytic activity [3]. Plasminogen binding to keratinocytes is reduced by TXA, which also prevents UV light‐induced plasmin activation. Tyrosinase activation in melanocytes is a crucial factor in skin hyperpigmentation, and it is triggered by plasmin activation, which also causes keratinocytes to secrete alpha‐mesh and inflammatory mediators such as prostaglandin E2 and alpha‐archiedonic acid. It is believed that TXA's antiangiogenic impact results from a reduction in plasmin‐mediated production of vascular endothelial growth factor and endothelin‐1, two important skin angiogenic factors implicated in the vascular hypothesis of melasma pathogenesis [4, 5].

Several reviews have explored tranexamic acid (TXA) in pigmentary disorders, primarily melasma and post‐inflammatory hyperpigmentation (PIH). Godse et al. reported oral TXA as effective and well‐tolerated for melasma [6], while Chen et al. extended its use to rosacea, Riehl's melanosis, and post‐acne erythema, emphasizing its anti‐melanogenic and anti‐inflammatory effects [7]. Konisky et al. found oral TXA most effective but associated with gastrointestinal and menstrual side effects, whereas intralesional and microneedling‐assisted topical TXA were safer alternatives [8]. Similarly, Alsharif et al. showed that all TXA routes benefit PIH, with intradermal delivery offering the best balance of efficacy, safety, and cost [9].

However, these reviews are limited by their focus on specific conditions such as melasma or PIH and do not comprehensively cover all hyperpigmentation disorders, including post‐traumatic pigmentation, lentigines, and drug‐induced hyperpigmentation. Additionally, few have compared TXA directly with other established or adjunctive therapies such as lasers, chemical peels, hydroquinone, or azelaic acid, leaving the relative efficacy and safety among modalities unclear. Therefore, the present review aims to comprehensively evaluate the efficacy and safety of tranexamic acid across a wider range of hyperpigmentation disorders, integrating evidence from various administration routes and comparing its outcomes with other therapeutic options to provide a clearer, evidence‐based understanding of TXA's role in hyperpigmentation management and to guide clinicians toward optimized treatment strategies.

## 2 Methodology

This narrative review was conducted to comprehensively evaluate the efficacy and safety of tranexamic acid (TXA) in hyperpigmentation disorders, particularly melasma and post‐inflammatory hyperpigmentation (PIH). A structured literature search was performed in PubMed, Scopus, and Google Scholar for studies published between 2010 and 2025 using the keywords: (“Tranexamic Acid” OR “TXA”) AND (“Melasma” OR “Hyperpigmentation” OR “Post‐inflammatory Hyperpigmentation” OR “PIH” OR “Pigmentary Disorders”) AND (“Efficacy” OR “Treatment” OR “Safety”). Additional articles were identified through manual reference screening. Studies were included if they evaluated TXA for melasma, PIH, or related pigmentary disorders, focusing on efficacy, safety, or comparisons with standard treatments such as hydroquinone, triple combination creams, or laser therapies. All study designs, including randomized controlled trials, observational studies, case reports, and reviews, were considered, while non‐English, non‐human, and unrelated studies were excluded. Data were extracted regarding study design, sample size, TXA formulation (oral, topical, intradermal), dosage, treatment duration, outcomes (MASI/mMASI reduction, pigmentation improvement, patient satisfaction), and adverse effects. Findings were synthesized descriptively and organized into comparative tables according to TXA route and comparator therapy. Given the heterogeneity of study designs and outcome measures, a qualitative rather than quantitative approach was applied. The analysis emphasized comparative efficacy, formulation‐specific outcomes, and safety.

Although not systematic, this review adhered to structured inclusion criteria and relied solely on peer‐reviewed literature to ensure scientific rigor and clinical relevance.

The eligibility criteria used in this review are summarized in Table 1.

**TABLE 1:** Eligibility criteria.

Inclusion criteria	Exclusion criteria
Studies evaluating TXA for melasma, PIH, or other hyperpigmentation disorders	Studies unrelated to pigmentary disorders
Articles focusing on *efficacy*, *safety*, or *comparison* with standard treatments (e.g., hydroquinone, laser therapy)	Non‐English language studies
All study designs: randomized controlled trials (RCTs), observational studies, case reports, reviews, and expert opinions	Studies on pediatric populations not involving hyperpigmentation
Human studies published in peer‐reviewed journals	Animal or in vitro studies

## 3 Results

### 3.1 Melasma

#### 3.1.1 Oral TXA

A competitive overview of melasma studies by route of administration is presented in Table 2.

**TABLE 2:** Comparative summary of studies on tranexamic acid (TXA) for melasma by route of administration.

Route of administration	Study design and sample size	Dose/Intervention	Duration	Main findings/Efficacy outcomes	Adverse effects/Safety
Oral TXA	Multiple RCTs and prospective trials (*n* = 37–132)	250–500 mg BID; some up to 1500 mg/day	8–24 weeks (some up to 6 months)	Significant reduction in MASI (up to 49%–95% improvement); sustained effect post‐therapy in moderate cases; enhanced quality of life; effective as mono or adjunct therapy	Generally well tolerated; mild GI upset, menstrual irregularities, headache; no major thromboembolic events reported
Topical TXA	RCT and prospective studies (*n* = 23–60)	TXA 2% emulsion/mask or 5% cream; compared to Hydroquinone 2%	12 weeks	Comparable MASI reduction to hydroquinone with fewer side effects; histologic improvement (↓melanin, ↓VEGF, ↓ET‐1); early visible improvement by week 4–8	Mild irritation; no systemic side effects
Intralesional TXA (Mesotherapy)	Split‐face RCTs and small trials (*n* = 5–60)	4 mg/mL TXA injected intradermally every 2–4 weeks	12–24 weeks (some with follow‐up to 48 weeks)	Significant reduction in mMASI and hemi‐mMASI; 60%–90% response rate; comparable to cysteamine or laser therapy; relapse in some cases at long‐term follow‐up	Injection site pain, burning, erythema, transient hypopigmentation; minimal systemic risk

The FDA presently approves oral TXA for haemoptysis (500 mg inhalation TID), cyclic heavy menstrual bleeding (1300 mg PO TID), and tooth extraction in hemophiliac patients (650 mg PO or injection TID). On the other hand, oral TXA is being used off‐label to treat melasma, especially in situations when traditional first‐line treatments are ineffective. The effectiveness and safety of tranexamic acid (TXA) in the treatment of melasma have been examined in a number of research studies and expert evaluations, underscoring TXA's developing status as a dependable treatment choice for hyperpigmentation disorders. Experts in Indian dermatology analyzed the increasing amount of data demonstrating oral TXA's efficacy as melasma monotherapy and adjuvant treatment in a consensus statement [6]. TXA's anti‐fibrinolytic qualities and capacity to prevent melanocyte activation make it a viable therapy choice, the panel underlined, especially for individuals who are not responding to conventional therapies. For best results, they advised cautious patient selection and proper dosage. Additional data was presented by Bala et al., who found in their thorough analysis that oral TXA, especially in Asian skin types, successfully lowers pigmentation in melasma at modest dosages (about 500 mg daily for 8–12 weeks) [10]. The review also affirmed its favorable safety profile, noting that TXA does not significantly increase thromboembolic risk when patients are appropriately screened for contraindications.

Thirteen individuals received a placebo for 12 weeks, whereas 20 patients received 250 mg TXA twice daily as part of a randomized controlled experiment. Using MASI, Melasma Quality of Life score, and colorimetry as metrics, 50% of patients receiving TXA had an improvement in melasma, compared to just 5.9% in the placebo group. There were no serious adverse effects noted [11]. Another randomized controlled trial of 39 participants compared oral TXA 250 mg twice daily with placebo for 12 weeks, followed by 12 weeks of sunscreen use. TXA produced a 49% mMASI reduction versus 18% in controls at 3 months, with sustained 26% improvement after discontinuation. Side effects, mainly gastrointestinal issues, menstrual changes, and headaches, were mild. The authors noted lasting benefits in moderate melasma but relapse in severe cases, suggesting longer treatment may be needed [12].

A multicenter trial of 72 severe melasma patients compared oral TXA doses (500–1500 mg) over 8 weeks plus a 2‐year follow‐up, showing no significant differences in MASI reduction or satisfaction among doses, despite a trend toward greater improvement at higher doses. Mild side effects included insomnia, stomach upset, and menstrual changes [13]. Another study of 132 patients found TXA 500 mg twice daily significantly reduced MASI scores and improved satisfaction faster than 250 mg once daily, with only minor transient side effects. The authors recommended initiating therapy with 500 mg twice daily, then maintaining with 250 mg once daily [14].

In a trial of 74 patients taking TXA 250 mg twice daily for 6 months, 94.6% showed improvement within 1–2 months and 95.9% achieved fair‐to‐excellent results by 6 months, with only seven relapses at follow‐up. Common side effects were mild GI upset and hypomenorrhea, with no coagulation changes [15]. Another study of 30 patients with refractory melasma treated with TXA 500 mg twice daily for 12 weeks found clinical improvement in all, with most showing moderate to good mMASI and quality‐of‐life scores. Histology revealed reduced pigmentation, melanocyte count, and inflammation, with effects maintained over 2 months [16].

#### 3.1.2 Topical TXA

For those who cannot handle oral TXA or who do not want to use oral treatment for melasma, topical TXA is a viable substitute since it delivers TXA directly to the skin. Comparing topical TXA to conventional topical therapy has been the subject of several investigations. 60 women participated in a 12‐week, randomized, double‐blind research that contrasted topical TXA 5% with HQ 2%. In addition to daily SPF 30, participants in both groups were told to apply the topical treatment twice daily to the afflicted regions. There was no discernible difference between the groups, despite the fact that both groups' MASI ratings dropped considerably. But compared to the HQ group, the TXA group reported fewer side effects and greater patient satisfaction [17].

In a different experiment, TXA 2% was applied as a sheet mask and an emulsion. For 12 weeks, participants were told to apply the mask three times a week and the emulsion twice a day. Between baseline and weeks four and eight, the mMASI score significantly decreased; however, between weeks 8 and 12, the outcomes were less significant. By the conclusion of 12 weeks, 22 of the 23 patients had shown a clinical improvement in their erythema and pigmentation. Fontana‐Masson staining of biopsies revealed a significant reduction in epidermal pigmentation. Furthermore, VEGF and endothelin‐1 showed a downward trend, indicating that topical TXA is useful in reducing melanogenesis and the number of vessels in the dermis [3].

#### 3.1.3 Intralesional TXA Injections

Mesotherapy, also known as intralesional injections, is the process of injecting TXA into the dermis using tiny needles, usually at a dosage of 4 mg/mL and spaced 1 cm apart. 4 mg/mL is the most often used concentration for intralesional therapy. It has been demonstrated that intralesional TXA is an effective treatment for melasma. Using an insulin syringe, 60 patients with moderate to severe melasma received injections of 4 mg/mL TXA on one side of their face and normal saline on the other side every 2 weeks for 12 weeks in a nonrandomized split‐face control experiment [18]. The TXA side of the face had a considerably lower Hemi‐mMASI score than the normal saline side at the 12‐week follow‐up, and this trend persisted at the 24‐week follow‐up. Ninety percent of patients showed a good to excellent response on the TXA side. Adverse effects of injected TXA include pain and burning at the injection sites, erythema, and hypopigmentation. On the TXA side, 90% of patients had satisfactory to outstanding responses. Erythema, hypopigmentation, discomfort, and burning at the injection sites are side effects of injected TXA.

The recurrence of melasma following intralesional injections was examined in one research study. Five individuals had seven sessions of 4 mg/mL TXA injections every 2 weeks, with a follow‐up period of 48 weeks (30‐gauge (G) needle). In week 16, the mMASI score significantly dropped. Nevertheless, by week 48, with a 60% recurrence rate, there was no statistically significant improvement in the mMASI score. From week 16 to week 48, patient satisfaction levels likewise declined. Despite the limited research size, the short‐term effectiveness of TXA injections is highlighted, and melasma has a high recurrence rate [19].

In a single‐blind RCT of 54 patients, 5% cream of cysteamine, an antioxidant and depigmenting molecule, was compared with 4 mg/mL TXA injections (insulin syringe with 28‐gauge needle). TXA injections were given every 4 weeks for 2 months, and cysteamine was administered every night for 4 months. According to the study, there were no appreciable differences between the two treatment groups, and both groups' mMASI scores significantly improved as compared to the baseline. However, as compared to the TXA injection group, the cysteamine group reported fewer adverse effects [20].

Intradermal platelet‐rich plasma (PRP) injections and 4 mg/mL TXA injections, administered every 4 weeks for 12 weeks (using an insulin syringe with a 30G needle), were compared in a nonrandomized controlled experiment. Both groups had a reduced mMASI score after 24 weeks, but the result, which was evaluated at that point, indicated a significant difference between the groups favoring the PRP group [21]. Even though PRP could be a more successful intradermal therapy, PRP injections are expensive, and additional research is needed to determine how beneficial they are. Split‐face research contrasted monthly treatment sessions with a maximum of four sessions (using an insulin syringe) using an Erbium: YAG laser with intradermal 4 mg/mL TXA injections. Although there was no discernible dermatoscopic change, the TXA therapy increased patient and physician satisfaction [22].

### 3.2 Post‐Inflammatory Hyperpigmentation (PIH)

A comparative summary of TXA studies for post‐inflammatory hyperpigmentation is presented in Table 3. Skin pigmentation resulting from trauma, surgery, or an inflammatory disease that affects both the epidermis and dermis is known as pigmentation of the skin (PIH). Patients of all skin tones can be affected by PIH; however, darker‐skinned groups including South Americans, Asians, and Africans are more prone to experience it. Topical medicines, such as hydroquinone (HQ), chemical peeling, laser treatments, and others are now used to treat PIH. Due to the possibility of adverse consequences including ochronosis, hydroquinone is prohibited for use in cosmetics [23]. As a safer non‐hydroquinone topical medication for PIH alone or supplementary treatment, TXA has drawn a lot of interest in recent years.

**TABLE 3:** Comparative efficacy and safety of tranexamic acid (TXA) for postinflammatory hyperpigmentation (PIH) by route of administration.

Route/Modality	Study design and population	Typical regimen/Dose	Main efficacy findings	Durability of response	Safety profile/Adverse effects
Oral TXA	Review, retrospective, and clinical prevention studies; 30–82 patients	250–750 mg 2–3× daily; up to 1500 mg/day	Consistently reduces PIH severity and promotes faster clearance; some studies show preventive benefit in high‐risk patients (Lindgren et al.); mixed results for post‐laser prevention (Japan, Thailand)	Moderate to high—improvements often persist weeks–months post‐therapy; prevention effect in long‐term users	Mild GI upset, menstrual irregularities, occasional nausea; no thromboembolic events reported
Topical TXA	Single‐blind and RCTs; 35–60 patients	2%–5% TXA serum/solution applied twice daily	Effective in improving skin tone, reducing dark spots and redness; comparable efficacy to azelaic acid; early visible lightening by 4–8 weeks	Moderate—benefits sustained with continued use; relapse possible after discontinuation	Excellent tolerability; occasional mild irritation or dryness
Intradermal/microinjection TXA	Split‐face and controlled lesion‐level studies; 25–40 participants	4–50 mg/mL injected every 2–4 weeks for 3 months	Significantly reduces PAHI and melanin index; may prevent laser‐induced PIH; results comparable to fractional laser but slower onset	Moderate—partial relapse may occur at 6–12 months if maintenance absent	Local pain, transient erythema, mild hypopigmentation; no systemic risks
Laser‐assisted topical TXA delivery (LADD)	Split‐area and retrospective studies; 10–25 patients	Topical 10% TXA with picosecond or fractional thulium laser	Mixed results—picosecond laser showed limited penetration (no added benefit), while fractional thulium laser improved drug absorption and clearance rates	Moderate—sustained improvement in 84% of cases (excellent/good clearance)	No major adverse events; minimal erythema or crusting
Combined TXA + Laser therapy	Case reports and meta‐analysis; 64‐year‐old woman, 7 RCTs (*n* = 222)	Oral TXA 750 mg/day + Q‐switched Nd: YAG; meta‐analysis: mixed routes	Case reports show marked and lasting clearance when combined; meta‐analysis found no significant long‐term benefit but short‐term MI improvement.	Variable — individual cases show durable results; pooled RCTs show only transient benefit.	Mild nausea, menorrhagia (oral TXA); laser‐related transient erythema.

#### 3.2.1 TXA Only or TXA vs. Other

TXA has garnered a lot of attention recently as a safer non‐hydroquinone topical drug for PIH either alone or in addition to other treatments [24]. With a favorable safety profile, Lindgren et al. used oral TXA 650 mg/day for periods ranging from 2 weeks to several years. The authors thought that TXA should be used as a therapy for all high‐risk patients before they received chemical peels, laser treatments, microneedling, cryolipolysis, cryohydrolysis, and cryotherapy [25]. Another study recruited 35 volunteers who received topical treatment with a novel face serum containing 2% TXA twice daily for 8 weeks. The findings demonstrated that the therapy was well tolerated, improved skin tone, and decreased face dark patches and redness in sun‐damaged skin [26]. Kim et al. [27] described a case in which oral and topical tranexamic acid (TXA) significantly improved post‐inflammatory hyperpigmentation (PIH). Following IPL and laser therapy, a 37‐year‐old lady acquired dark brown malar pigmentation. Three times a day, she took 250 mg of oral TXA, and three times a week, she got topical TXA wet dressings (15 mL of 500 mg/5 mL solution) for 20 min. The pigmentation significantly faded in just 2 weeks, leaving just faint pinkish spots [27]. A 12‐week single‐blind randomized clinical experiment was conducted by Sobhan et al. on patients with post‐acne PIH. Two groups of patients (30 in each group) received twice‐daily applications of 20% azelaic acid (AZA) cream and 5% TXA solution, respectively. TXA exhibited a higher safety profile in the first month, but both groups improved their post‐acne hyperpigmentation index (PAHI) ratings with similar effectiveness [28]. In a split‐face trial, Tawfic et al. compared low‐power fractional CO_2_ lasers (every 4 weeks) and TXA microinjections (every 2 weeks) for 3 months in 25 patients with refractory post‐acne hyperpigmentation. Both considerably lowered melanin index and PAHI scores; however, the CO_2_ laser produced a larger overall dermatoscopic improvement [29].

#### 3.2.2 TXA Combined With Other Treatment Methods

Following a month of unsuccessful usage of a hydroquinone cream, a 64‐year‐old lady with post‐inflammatory hyperpigmentation brought on by allergic contact following frequent use of pure henna hair color was treated with a low‐fluence 1064 nm Q‐switched Nd: YAG laser once a week and orally 750 mg TXA daily. The forehead's hyperpigmentation considerably decreased after 10 weeks, and the results continued to hold up at the 1‐year mark [30]. Ten patients with post‐traumatic PIH participated in randomized split‐area research that compared the application of 10% topical TXA using a picosecond laser alone with laser assistance. There were no discernible variations in the improvement or satisfaction of hyperpigmentation between the two groups, who had four treatments spaced 6 weeks apart. The incapacity of the picosecond laser to produce sufficient microchannels for drug penetration was the scientists' explanation for LADD's poor effectiveness [31]. Twenty‐five PIH patients (mean = 3.3 sessions) were studied retrospectively using a low‐energy 1927 nm fractional thulium fiber laser as an adjunct to LADD for topical TXA. No serious side effects or deterioration were seen, and the results revealed 16% complete, 68% excellent, and 16% good clearance. TXA penetration was improved, and heat damage was reduced by the laser's tiny, superficial channels, increasing effectiveness without increasing danger [32].

#### 3.2.3 Preventing PIH After Laser Therapy

PIH is more common in Asians following Q‐switched laser treatment, particularly Q‐switched ruby laser (QSRL) treatment. Clinical studies have also been conducted to see whether TXA helps prevent or lessen this type of PIH, but the results have been mixed. Following QSRL for the treatment of senile lentigines on the face, 32 Japanese women received oral TXA at a dose of 750 mg/day. However, the incidence of PIH did not substantially differ between the groups who got oral TXA and those that did not [33]. According to the findings of another trial conducted in Thailand, oral TXA (1500 mg/day) was administered beginning the day following Q‐switched 532‐nm Nd therapy. The oral TXA group showed a significantly lower incidence of pigmented granules at weeks 6 and 12 under dermatoscopy, indicating that oral TXA enhanced PIH clearance, although YAG laser therapy for solar lentigines was unsuccessful in preventing PIH after laser treatment [34]. Other researchers evaluated the impact of intradermal TXA injection in preventing PIH following solar lentigo therapy using a 532 nm Nd: YAG laser. Using a drawing technique, the lesions were randomized to the TXA group (intradermal injection of 50 mg/mL TXA) and the control group (intradermal injection of 0.9% normal saline). A broad‐spectrum sunscreen was given for 12 weeks after the crusts peeled off. At the conclusion of the trial, the TXA group had a lower incidence of PIH than the control group (16% vs. 28%), according to the data [35].

A systematic study and meta‐analysis evaluating the effectiveness of tranexamic acid (TXA) in reducing post‐inflammatory hyperpigmentation (PIH) after laser therapy was carried out by Feng et al. Although there was a temporary improvement after 1 month that favored TXA usage (*p* = 0.04), the results did not demonstrate a significant overall difference in the lowering of the melanin index (MI) between groups (*p* = 0.45). There were no discernible variations between the various TXA administration routes or during subsequent follow‐ups. With oral TXA, mild side effects such as nausea and menorrhoea were documented [36].

#### 3.2.4 Other Hyperpigmentation Disorders

##### 3.2.4.1 Infraorbital Hyperpigmentation

Numerous causes contribute to periorbital hyperpigmentation, a common cosmetic problem that frequently necessitates combined therapy. In order to examine the effectiveness of fractional CO_2_ laser and microneedling in combination with topical tranexamic acid (TXA), Ghandehari et al. undertook randomized research with 30 subjects who had infraorbital hyperpigmentation. Following three monthly treatment sessions, a cotton swab was used to apply 2 cc of TXA (500 mg/5 mL) to the treated region. With no discernible negative effects, both groups demonstrated a considerable improvement over baseline. At days 30, 90, and 150, there was no discernible difference between the two modalities; however, on day 60, the fractional CO_2_ laser + TXA group showed marginally better outcomes, indicating increased early efficacy without sacrificing safety [37].

##### 3.2.4.2 Riehl's Melanosis

There are few effective therapies for Riehl's melanosis, an acquired pigmentation illness that manifests as brown or blue discolouration on the forehead, temples, and zygomatic regions. Post‐inflammatory hyperpigmentation (PIH) is a danger associated with laser treatment alone, particularly in darker skin types. When used in conjunction with laser therapy, TXA's anti‐melanogenic, anti‐angiogenic, and anti‐inflammatory qualities may improve results and lower the risk of PIH. Kwon et al. used oral TXA 250 mg daily, nightly 4% hydroquinone cream, and a very low‐fluence 1064‐nm Q‐switched Nd: YAG laser (10–18 sessions) to treat eight patients with refractory Riehl's melanosis. Three patients had “almost clear” results after therapy, while five had “marked improvement,” with notable decreases in erythema and melanin indices proving the regimen's effectiveness and safety [38]. The safety and efficacy of oral TXA and glycyrrhizin compound in treating intractable Riehl's melanosis were evaluated by Xu et al. Ten participants in the trial received daily 500 mg doses of TXA plus 150 mg of the chemical glycyrrhizin for 3 months, followed by 3 months of TXA daily alone. As demonstrated by reflectance confocal microscopy (RCM) and dermoscopy studies, seven patients had a substantial improvement in their symptoms, with a significant decrease in both MI and erythema indices (EI) [39]. In order to assess the effectiveness of oral TXA alone and oral TXA coupled with IPL in treating refractory Riehl's melanosis, the same research team subsequently conducted a prospective trial with 28 patients. For 6 months, 11 of the patients got monthly IPL treatment in addition to 500 mg of oral TXA daily. Following therapy, both groups' mean MI, EI, and dermal macular hyperpigmentation area and severity index (DPASI) considerably decreased, with the combination group seeing a more positive outcome [40].

##### 3.2.4.3 Macular Amyloidosis (MA)

Brown, reticulated, hyperpigmented macules are the hallmark of macular amyloidosis (MA), a prevalent kind of cutaneous amyloidosis that frequently defies typical therapies like topical corticosteroids or retinoids. 43 MA patients were split into two groups for a randomized study by Ghassemi et al. One group received intradermal injections of TXA (4 mg/mL) every 2 weeks for six sessions, while the other group applied a topical Kligman combination (a mixture of 500 mg vitamin C, 0.05% tretinoin, 0.01% fluocinolone acetonide, and 4% hydroquinone) every night for 12 weeks. Although hyperpigmentation and symptom alleviation were significantly improved in both groups, the TXA injection group's melanin content was reduced more, suggesting that TXA is more effective in treating MA‐related pigmentation [41].

##### 3.2.4.4 Lichen Planus Pigmentosus

Clinical data from 17 individuals with lichen planus pigmentosus were gathered in Moroccan research; 11 of these patients received oral TXA 500 mg twice daily for 3 or 6 months. High‐potency corticosteroids and hydroquinones were also administered to all but one patient. Only four patients had full resolution of the lesions throughout the follow‐up; the lesions in the other patients stayed the same [42].

### 3.3 Comparison of TXA With Other Therapies

#### 3.3.1 Tranexamic Acid (TXA) vs. Hydroquinone (HQ)

In a Moroccan study, clinical data was collected from 17 patients with lichen planus pigmentosus, 11 of whom were given oral TXA 500 mg twice daily for 3 or 6 months. All but one patient additionally received hydroquinones and high‐potency corticosteroids. During the follow‐up period, the lesions in only four patients completely disappeared; the lesions in the remaining patients remained unchanged [43]. Comparing topical 5% TA solution with 3% HQ cream for 12 weeks in 100 patients, an Indian RCT found that while MASI reductions were almost comparable (TA = 27% vs. HQ ≈ 26.7%), patient satisfaction and TA tolerability were greater [44]. Intradermal TXA injections and HQ 4% cream at several concentrations were contrasted in a separate split‐face investigation. The low‐dose TA (4 mg/mL) was less successful at 12 weeks, indicating a dose‐dependent response, whereas the high‐dose TA (10 mg/mL) group saw improvement equivalent to HQ [45]. Along with improved safety and patient acceptability with TA, a more recent RCT comparing topical 3% TA versus 4% HQ cream also showed comparable decreases in melasma severity [46].

#### 3.3.2 TXA + Hydroquinone Combination vs. Hydroquinone Alone

Combination therapy appears to enhance depigmenting efficacy. Local infiltration (intradermal) TXA + 4% HQ cream was compared to HQ alone for 12 weeks in a split‐face, prospective RCT including 55 participants. Patient satisfaction and MASI decrease were considerably higher on the TXA + HQ side, indicating an additive impact [47]. Oral TXA (250 mg BID) in combination with HQ 4% + sunscreen was compared to HQ + sunscreen + placebo by Shihab et al. [48]. After 3 months, the combination group's mMASI decrease was around 55%, whereas the control groups were approximately 10.9%. Three months after stopping, benefits continued, but there was a slight relapse [48].

#### 3.3.3 TXA vs. Triple Combination Cream (Hydroquinone + Tretinoin + Fluocinolone)

Although TXA has been compared with triple combination cream (TCC), TCC is still the gold standard topical treatment. 150 melasma patients were randomly assigned to receive intradermal TXA or TCC (HQ 4% + tretinoin 0.05% + fluocinolone 0.01%) in comparative research conducted in Pakistan. The authors concluded that both were successful, with fewer side effects and higher tolerance for TXA, even if numerical data indicated a bigger MASI decrease with TCC (−6.1 vs. −1.9) [49]. In different research, topical TCC and TXA mesotherapy (50 mg/mL every 2 weeks) were compared over 12 weeks. The TXA group saw a 44.4% MASI decrease, whereas the TCC group experienced a 34.2% reduction (*p* = 0.08). TXA has shown encouraging effectiveness and fewer side effects, albeit being statistically borderline [50]. In a randomized controlled trial, oral TXA (325 mg BID) and TCC cream were found to reduce MASI more quickly and effectively than oral TXA alone, especially at early (4–8 week) intervals [51].

#### 3.3.4 TXA vs. Intense Pulsed Light (IPL) and Laser Therapies

Intradermal TXA has been compared to light‐based devices in comparative experiments. While there was no discernible difference in the final mMASI scores, research comparing intradermal TXA and intense pulsed light (IPL) therapy over a 6‐month period indicated that both modalities considerably decreased the severity of melasma. TXA demonstrated consistent improvement and fewer adverse effects, although IPL acted more quickly at first [52]. In split‐face research, the combined arm showed higher dermoscopic improvement and larger MASI reduction when comparing Q‐switched Nd: YAG laser + intradermal TXA versus TXA alone [53]. Without increasing adverse effects, a meta‐analysis conducted in 2023 found that laser + topical TXA combination treatment worked much better than laser alone (*p* < 0.0001), particularly when fractional CO_2_ laser + frequent topical TXA was used [54]. Another systematic analysis of oral TXA + laser found 12%–15% greater mMASI reduction and lower recurrence (< 20% at 6 months) versus laser alone [55]. A competitive evaluation of TXA versus other melasma therapies is summarized in Table 4.

**TABLE 4:** Comparative efficacy and safety of tranexamic acid (TXA) versus other melasma therapies.

Comparator therapy	Key studies (Ref)	Study design/population	TXA regimen (route and dose)	Comparator regimen	Main efficacy findings	Safety/tolerability
Hydroquinone (HQ)	[43, 44, 45, 46]	Multiple RCTs and split‐face trials (*n* = 100–200, female predominance)	Topical 3%–5% TXA cream or intradermal 4–10 mg/mL injections for 12 weeks	HQ 3%–4% cream	Both TXA and HQ achieved similar MASI reduction (~ 25%–30%); histology favored TXA (↓melanin area%); dose‐dependent response for intradermal TXA	TXA caused fewer reactions (erythema, irritation) and higher satisfaction; HQ had more local irritation
TXA + HQ combination vs. HQ alone	[47, 48]	Split‐face and double‐blind RCTs (*n* = 55–120)	Intradermal TXA 4 mg/mL + HQ 4% cream; Oral TXA 250 mg BID + HQ + sunscreen	HQ 4% + sunscreen ± placebo	Combination achieved significantly higher MASI/mMASI reduction (~ 55% vs. 10%–12%); improved satisfaction; effect persisted 3 months post‐treatment	Mild GI upset, transient headache, no major events
Triple combination cream (HQ + tretinoin + fluocinolone)	[49, 50, 51]	RCTs and comparative clinical trials (*n* = 150+)	Intradermal TXA (50 mg/mL q2weeks) or Oral TXA 325 mg BID ± TCC	TCC (HQ 4% + Tretinoin 0.05% + Fluocinolone 0.01%)	Both improved melasma; TCC often showed faster onset, but TXA had fewer side effects. TXA mesotherapy achieved 44% vs. 34% MASI reduction (NS). Oral TXA + TCC combo gave faster results than TXA alone	TXA caused fewer steroid‐related and irritant effects; well tolerated orally and intradermally
IPL and laser‐based therapies	[52, 53, 54, 55]	Split‐face RCTs, meta‐analyses (*n* = > 300 total)	Intradermal TXA (4 mg/mL q2weeks) or topical TXA with laser; Oral TXA + laser	IPL, Q‐switched Nd:YAG, and fractional CO_2_ laser alone	TXA alone ≈ IPL efficacy with slower onset but fewer recurrences. Laser + TXA (topical/oral) outperformed laser alone by 12%–15% MASI gain; lower relapse (< 20% at 6 months)	Laser‐related erythema and crusting only; systemic TXA mild GI or menstrual effects
Azelaic acid (AZA)	[28] (cross‐reference)	Single‐blind RCT, 60 PIH patients	Topical 5% TXA solution	AZA 20% cream	Both groups showed comparable PAHI improvement; TXA was safer in first month with less irritation	Mild irritation with AZA > TXA

## 4 Conclusion

Tranexamic acid (TXA) has emerged as an effective, safe, and versatile agent for managing hyperpigmentation disorders, particularly melasma and post‐inflammatory hyperpigmentation. Across various formulations—oral, topical, and intradermal—TXA consistently demonstrates significant pigment reduction, improved patient satisfaction, and a favorable safety profile with minimal adverse effects. When used alone or in combination with standard therapies such as hydroquinone, triple‐combination creams, or laser treatments, TXA enhances treatment efficacy and reduces relapse risk. Its multimodal mechanisms—antimelanogenic, anti‐inflammatory, and antiangiogenic—make it uniquely beneficial in addressing the complex pathophysiology of pigmentary disorders. Future large‐scale, long‐term randomized trials are warranted to establish standardized dosing regimens, clarify relapse prevention, and further define TXA's role as a cornerstone therapy in hyperpigmentation management. Tranexamic acid demonstrates consistent efficacy and safety across multiple routes of administration in hyperpigmentation disorders. Its multimodal mechanism supports its use as an emerging therapeutic option in dermatologic pigment management.

## Author Contributions

Dr. Ahmed AlJabr: conceptualization, clinical supervision, manuscript review. Aseel Muhana I. AlAnazi: literature search, data extraction, drafting sections of the manuscript. Rakan Abdulkarim A. AlEtebi: writing, evidence synthesis, manuscript structuring, final editing, corresponding author duties.

## Funding

This authors have nothing to report.

## Ethics Statement

This authors have nothing to report.

## Conflicts of Interest

The authors declare no conflicts of interest.

## Data Availability

The data that support the findings of this study are available from the corresponding author upon reasonable request.
